# Enhanced behavioral responses to cold stimuli following CGRPα sensory neuron ablation are dependent on TRPM8

**DOI:** 10.1186/1744-8069-10-69

**Published:** 2014-11-19

**Authors:** Eric S McCoy, Mark J Zylka

**Affiliations:** Department of Cell Biology and Physiology, UNC Neuroscience Center, The University of North Carolina, CB #7545, Chapel Hill, North Carolina 27599 USA

**Keywords:** Pain, Nociception, TRPM8, TRPV1, Thermoregulation

## Abstract

**Background:**

Calcitonin gene-related peptide-α (CGRPα) is a classic marker of peptidergic nociceptive neurons and is expressed in myelinated and unmyelinated dorsal root ganglia (DRG) neurons. Recently, we found that ablation of *Cgrpα*-expressing sensory neurons reduced noxious heat sensitivity and enhanced sensitivity to cold stimuli in mice. These studies suggested that the enhanced cold responses were due to disinhibition of spinal neurons that receive inputs from cold-sensing/TRPM8 primary afferents; although a direct role for TRPM8 was not examined at the time.

**Results:**

Here, we ablated *Cgrpα*-expressing sensory neurons in mice lacking functional TRPM8 and evaluated sensory responses to noxious heat, cold temperatures, and cold mimetics (acetone evaporative cooling and icilin). We also evaluated thermoregulation in these mice following an evaporative cold challenge. We found that ablation of *Cgrpα*-expressing sensory neurons in a *Trpm8*^*-/-*^ background reduced sensitivity to noxious heat but did not enhance sensitivity to cold stimuli. Thermoregulation following the evaporative cold challenge was not affected by deletion of *Trpm8* in control or *Cgrpα*-expressing sensory neuron-ablated mice.

**Conclusions:**

Our data indicate that the enhanced behavioral responses to cold stimuli in CGRPα sensory neuron-ablated mice are dependent on functional TRPM8, whereas the other sensory and thermoregulatory phenotypes caused by CGRPα sensory neuron ablation are independent of TRPM8.

**Electronic supplementary material:**

The online version of this article (doi:10.1186/1744-8069-10-69) contains supplementary material, which is available to authorized users.

## Introduction

Somatosensory stimuli are detected by neurons located in the dorsal root ganglia (DRG), which then transmit this information to the spinal cord for processing [1]. In the DRG, nociceptive neurons sense noxious thermal and mechanical stimuli and are broadly characterized as peptidergic or non-peptidergic, with peptidergic neurons commonly marked by calcitonin gene-related peptide immunoreactivity (CGRP-IR). CGRP-IR reflects expression of two closely linked genes, *Cgrpα* (*Calca)* and *Cgrpβ* (*Calcb)*. Both genes are expressed in DRG neurons; although *Cgrpα* is expressed at higher levels [2].

We recently generated a mouse line in which *Cgrpα*–expressing sensory neurons could be ablated in an inducible fashion, thus allowing us to examine how somatosensation is impaired in the absence of these neurons [3]. To accomplish this, we crossed mice with a LoxP-stopped human diphtheria receptor (hDTR) knocked-in to the *Cgrpα* locus with *Advillin-Cre* mice, generating “*Cgrpα-DTR*^*+/-*^” mice [3]. *Advillin-Cre* was used to restrict hDTR expression to *Cgrpα*-expressing sensory neurons [3–5]. CGRPα sensory neurons were then ablated in adult mice following two sequential intraperitoneal (i.p.) injections of diphtheria toxin (DTX). Following ablation, we found that sensitivity to noxious heat, capsaicin, and two pruritogens (histamine and chloroquine) were greatly reduced and thermoregulation following an evaporative cold challenge was impaired. Mechanical sensitivity was unaffected. In contrast, CGRPα sensory neuron-ablated mice showed enhanced behavioral responses to cold stimuli and cold mimetics, including enhanced responses to icilin at a dose that selectively activates TRPM8 *in vivo* [6]. This enhanced responsiveness was not due to a change in the number of TRPM8 immunoreactive (TRPM8^+^) DRG neurons or to increased peripheral nerve responses to cold stimuli. Instead, our findings suggested that CGRPα primary afferents cross-inhibited postsynaptic spinal neurons that were cold/icilin sensitive. Thus, ablation of CGRPα sensory neurons led to disinhibition of spinal neurons that were postsynaptic to TRPM8 afferents, suggesting a spinal mechanism for enhanced cold responsiveness. Moreover, our data suggested that TRPM8 activity might be responsible for driving enhanced cold sensitivity when CGRPα sensory neurons were ablated, although we did not test this possibility directly.

Here, we sought to directly evaluate whether enhanced cold sensory responses in CGRPα sensory neuron-ablated mice were dependent on TRPM8. To accomplish this, we crossed *Cgrpα-DTR*^*+/-*^ mice with *Trpm8*^*-/-*^ mice to produce “*DTR-Trpm8*^*-/-*^” mice. With these mice, we were able to inducibly ablate CGRPα sensory neurons in a genetic background that lacks functional TRPM8. We then compared somatosensory responses in these mice, pre- and post-ablation, to *Cgrpα-DTR*^*+/-*^ mice—mice with functional TRPM8.

## Results and discussion

### The total number of NF200^+^ DRG neurons is reduced following CGRPα sensory neuron ablation

**Figure 1:**
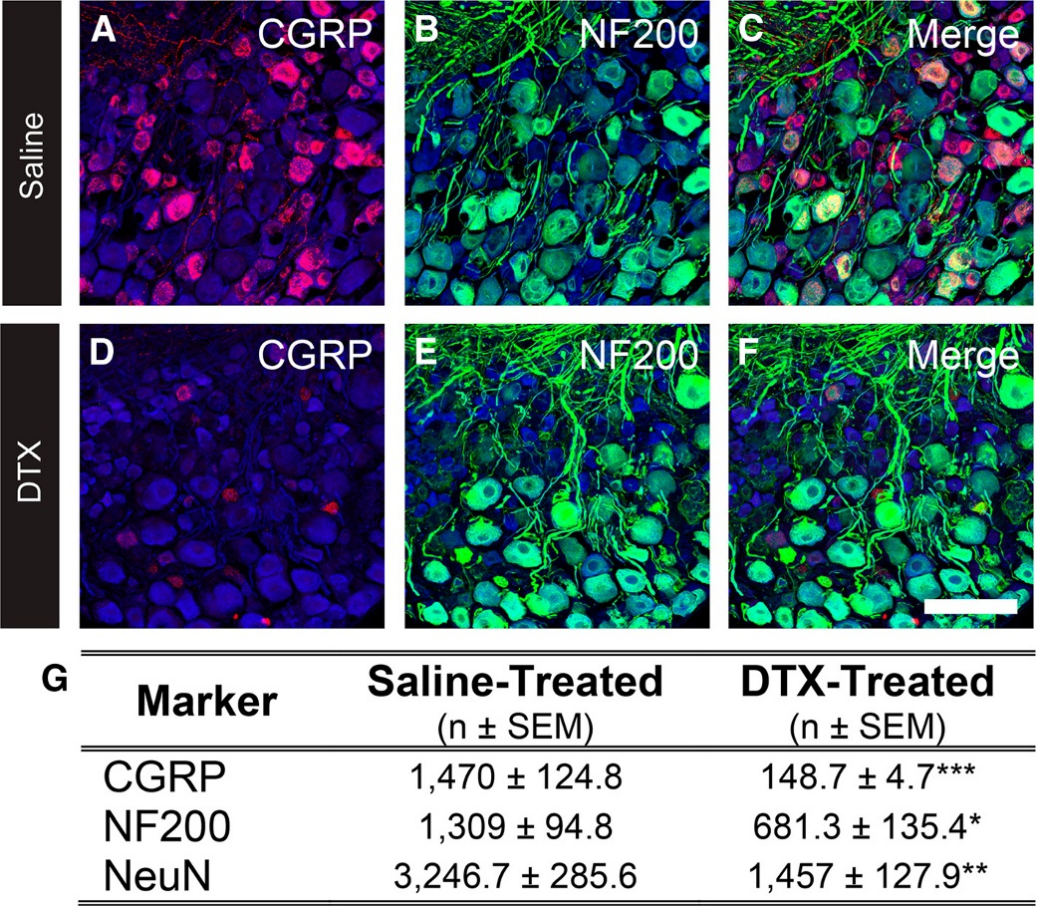
**Ablation of CGRPα sensory neurons reduces the number of NF200-expressing DRG neurons. (A-F)** Lumbar DRG from *Cgrpα-DTR* ^*+/-*^ mice treated with saline or DTX were stained with antibodies to CGRP (**A** and **D**, red), NF200 (**B** and **E**, green), and NeuN (**A**-**F**, blue). **(C and F)** Merged images. Images were acquired by confocal microscopy. Scale bar in F is 100 μm. **(G)** Quantification of the number of cells stained for CGRP and NF200. Cell counts are representative of n = 3 mice per treatment group, mean ± SEM. *p <0.05, **p <0.005, ***p <0.0005. Tissue was collected 7 days after the second saline/DTX injection.

In our previous study, we assessed the extent to which different classes of DRG neurons were ablated in our *Cgrpα-DTR*^*+/-*^ mice by quantifying the number of marker-positive neurons relative to NeuN^+^ neurons. We did not detect a significant change in the percentage of NF200^+^ neurons in CGRPα sensory neuron-ablated mice relative to saline-injected controls [3], despite the fact that 24% of all *Cgrpα*-expressing neurons expressed NF200 [7]. We speculated that this discrepancy might reflect our use of NeuN to normalize neuron counts [3]. Thus, to reexamine the extent to which NF200^+^, presumably myelinated, neurons are ablated, we treated additional *Cgrpα-DTR*^*+/-*^ mice with saline or DTX (n = 3 / group), sacrificed the mice 7 days later, then immunostained stained every fourth section through the L4 DRG for CGRP, NF200 and NeuN. We then imaged each section in its entirety by confocal microscopy and counted every marker-positive neuron (Figure 1A-F). This allowed us to quantify the absolute number of marker-positive neurons (Figure 1G). Based on these counts, we detected a 90% reduction in the number of CGRP-IR neurons in DTX-treated mice, confirming that CGRP-IR neurons were largely eliminated. There was also a significant (~50%) reduction in the number of NF200^+^ neurons in DTX-treated mice. Notably however, the percentage of NF200 neurons was not significantly different (40.3% in saline-treated versus 46.7% in DTX-treated; 1,309/3,246 versus 681/1,457, respectively), as we previously reported [3]. Thus, use of absolute cell counts provides a more accurate estimate of how many cells of a given type are ablated, although determining absolute cell numbers requires significantly more time and effort. Moreover, these data suggest that the enhanced cold sensory phenotype in CGRPα sensory neuron-ablated mice could reflect a loss of unmyelinated and/or myelinated CGRPα neurons.

### The enhanced behavioral responses to cold stimuli in CGRPα sensory neuron-ablated mice are dependent on TRPM8

TRPM8 neurons respond to cold stimuli, including cold mimetics like icilin, and are expressed in myelinated or unmyelinated neurons [8, 9]. We previously found that ablation of *Cgrpα*-expressing DRG neurons resulted in enhanced sensitivity to cold stimuli, including cold mimetics like icilin. Moreover, following ablation, there was increased activity in spinal neurons that are postsynaptic to icilin-responsive, TRPM8^+^ primary afferents [3]. These data suggested that the enhanced sensitivity to cold stimuli might be driven by enhanced tonic and evoked activity in TRPM8^+^ neurons. To test this possibility, we took advantage of the fact that tonic and evoked activity in TRPM8 neurons can be greatly reduced by deleting *Trpm8* [10]. Thus, to directly assess whether the enhanced cold sensory responses were TRPM8-dependent, we introduced the *Trpm8*^*-/-*^ allele into our *Cgrpα-DTR*^*+/-*^ line (see Methods for details), generating *DTR-Trpm8*^*-/-*^ mice. We then behaviorally tested these mice, along with *Cgrpα-DTR*^*+/-*^ mice (positive controls), before and after ablating CGRPα sensory neurons (by injecting 100 μg/kg DTX; two i.p. injections separated by 72 h). Additional groups of mice were injected with saline and served as controls.

**Figure 2:**
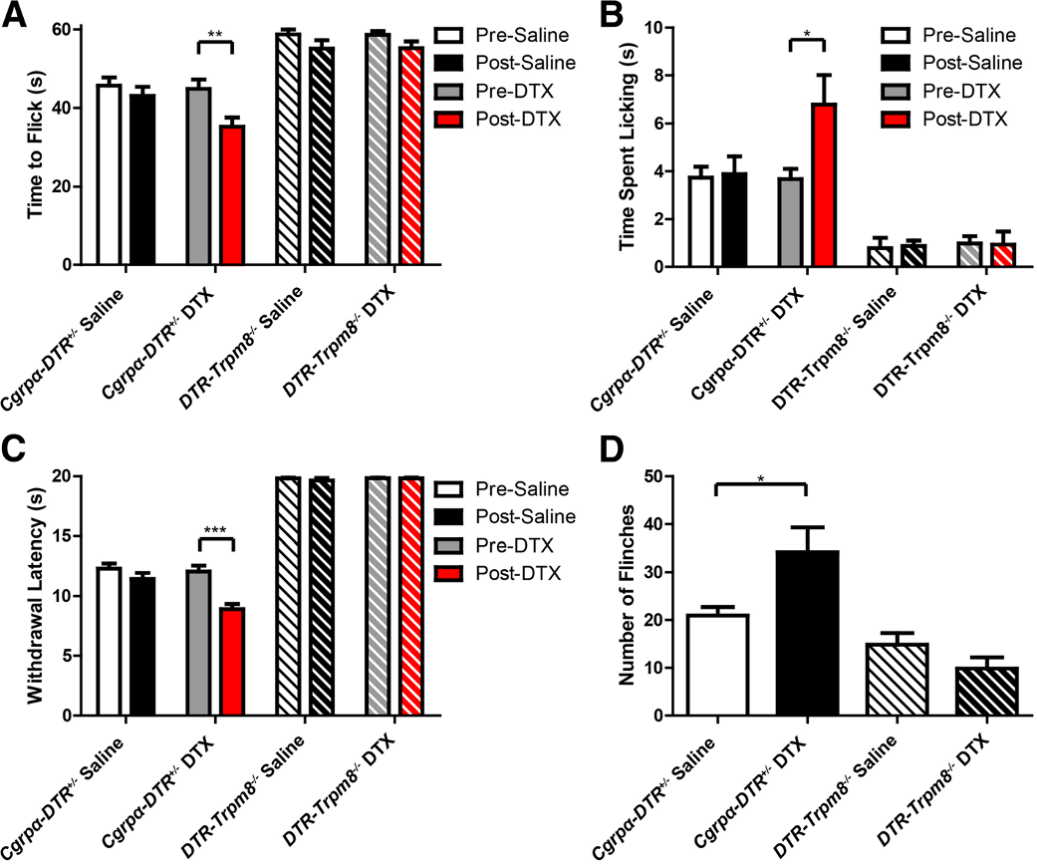
**Enhanced behavioral responses to cold stimuli in CGRPα sensory neuron-ablated mice is dependent on TRPM8.** Saline- and DTX-treated *Cgrpα-DTR* ^*+/-*^ and *DTR-Trpm8* ^*-/-*^ mice were tested in the **(A)** -10°C tail withdrawal assay, **(B)** acetone-(50 μl) evoked evaporative cooling assay, **(C)** cold plantar assay, and **(D)** after injecting icilin (2.4 μg/μl) into the hindpaw. The number of flinches that occurred over 10 min was measured. **(A-D)** t-tests were used to compare pre-saline/DTX and post-saline/DTX-treated mice. All values represent mean ± SEM. *p < 0.05, **p < 0.005, ***p < 0.0005. In **(B)**, all TRPM8^-/-^ mice showed significantly less time licking hindpaw in the acetone-evoked evaporative cooling assay compared to WT controls (p < 0.0005).

First, these mice were tested using a variety of cold-related behavioral assays (Figure 2). Consistent with our previous study [3], and as positive controls, DTX-treated *Cgrpα-DTR*^*+/-*^ mice showed enhanced cold sensitivity in the cold tail immersion assay (as evidenced by a reduction in the latency to flick the tail after DTX treatment; Figure 2A), enhanced sensitivity to acetone-evoked evaporative cooling (Figure 2B), enhanced sensitivity in the cold plantar assay (Figure 2C) and enhanced sensitivity following injection of icilin into the hindpaw (Figure 2D). In contrast, saline- and DTX-treated *DTR-Trpm8*^*-/-*^ mice were less sensitive to each of these cold stimuli, consistent with the observation that *Trpm8*^*-/-*^ mice are less sensitive to cold stimuli [11, 12]. Intriguingly however, DTX-treated *DTR-Trpm8*^*-/-*^ mice did not show enhanced responses to cold stimuli relative to saline-treated *DTR-Trpm8*^*-/-*^ mice, suggesting that the enhanced cold sensory responses following CGRPα sensory neuron ablation are TRPM8-dependent.

**Figure 3:**
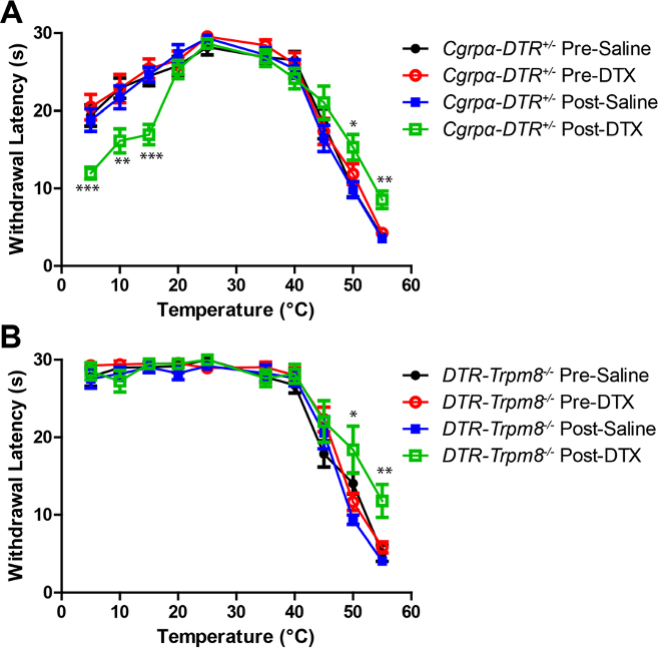
**Ablation of CGRPα sensory neurons in** ***DTR-Trpm8*** ^***-/-***^ **mice reduces heat sensitivity but does not enhance cold sensitivity.** Sensitivity to temperatures ranging from noxious hot to noxious cold was measured using the hindpaw withdrawal assay. **(A)** *Cgrpα-DTR* ^*+/-*^ and **(B)** *DTR-Trpm8* ^*-/-*^ mice pre- and post-saline/DTX treatment. All values are represented as mean ± SEM. Cutoff time was 30 s. Animals that did not withdraw their paw by this cutoff time were assigned a cutoff time of 30 s. t-tests were used to compare time points between pre-saline/DTX and post-saline/DTX-treated mice. *p < 0.05, **p < 0.005, ***p < 0005.

Next, we measured hindpaw withdrawal latency to temperatures ranging from noxious cold (5°C) to noxious hot (55°C) pre- and post-ablation. Consistent with our previous study, DTX-treated *Cgrpα-DTR*^*+/-*^ mice were more sensitive to noxious cold temperatures (5°C, 10°C, 15°C) and less sensitive to noxious hot temperatures (50°C and 55°C; Figure 3A). Likewise, DTX-treated *DTR-Trpm8*^*-/-*^ mice were less sensitive to noxious hot temperatures in this assay (Figure 3B). However, *DTR-Trpm8*^*-/-*^ mice were insensitive to all noxious cold temperatures, and cold sensitivity was not enhanced after DTX-treatment (Figure 3B).

**Figure 4:**
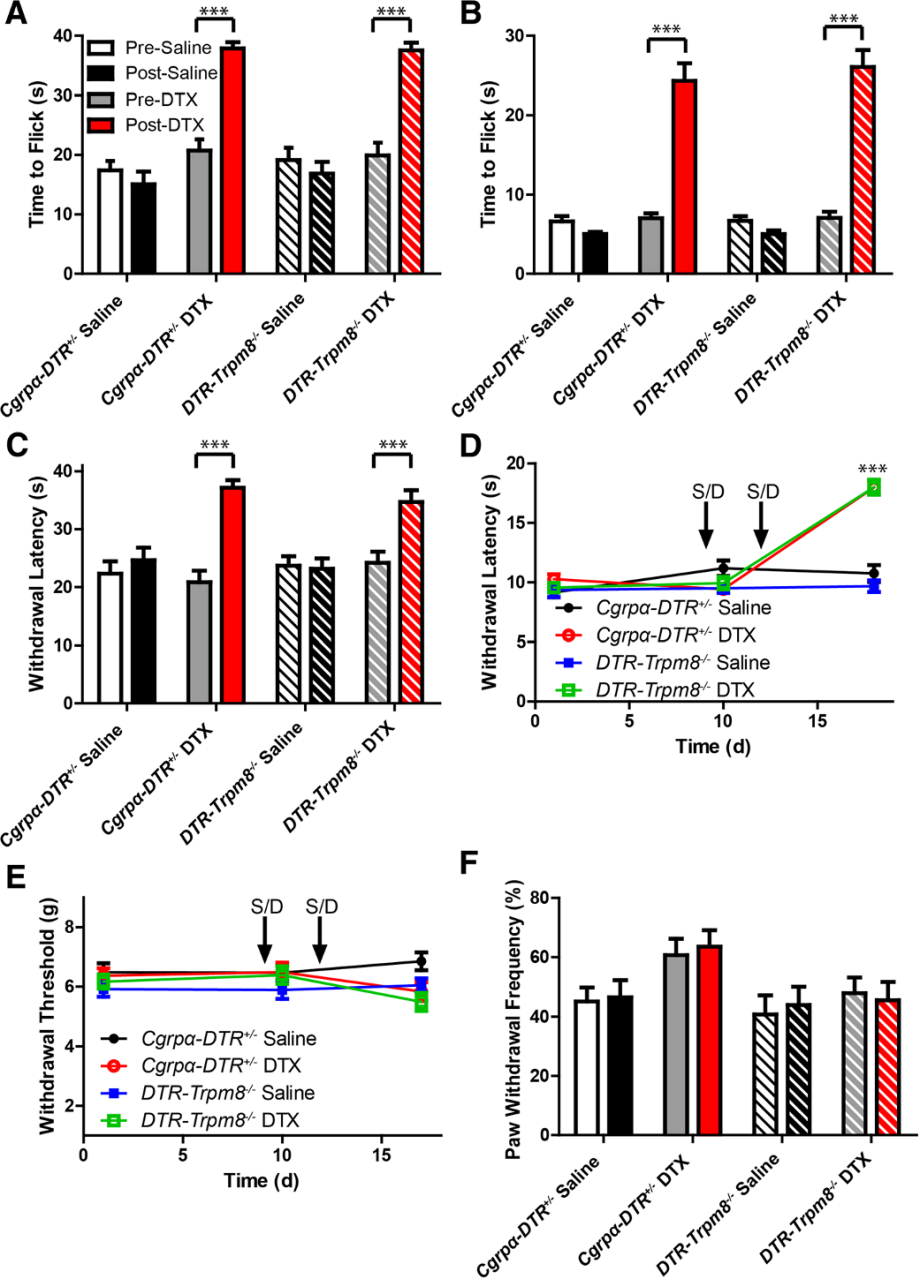
**Ablation of CGRPα sensory neurons in** ***DTR-Trpm8*** ^***-/-***^ **mice reduces heat sensitivity without affecting mechanical sensitivity.** Saline- and DTX-treated *Cgrpα-DTR* ^*+/-*^ and *DTR-Trpm8* ^*-/-*^ mice were tested in the **(A)** tail immersion assay at 46.5°C, **(B)** tail immersion assay at 49°C, **(C)** 52°C hot plate assay, **(D)** Hargreave’s assay (radiant heating of the hindpaw), and **(E)** electronic von Frey assay (to measure mechanical sensitivity). **(F)** Innocuous mechanical sensitivity was measured using the cotton swab assay. All values are represented as mean ± SEM. t-tests were used to compare time points between pre-saline/DTX and post-saline/DTX-treated mice. *p < 0.05, **p < 0.005, ***p < 0005.

Consistent with our previous study, DTX-treated *Cgrpα-DTR*^*+/-*^ mice were less sensitive to other noxious heat stimuli, including tail immersion in 46.5°C and 49°C water (Figure 4A,B), a hot plate set at 52°C (Figure 4C) and radiant heating of the hindpaw (Figure 4D; pre- and post-ablation). Likewise, DTX-treated *DTR-Trpm8*^*-/-*^ mice were less sensitive to noxious heat stimuli relative to saline-treated controls (Figure 4A-D). Mechanical sensitivity (assessed with electronic von Frey apparatus; Figure 4E) and innocuous mechanical sensitivity (Figure 4F) were not affected in DTX-treated *Cgrpα-DTR*^*+/-*^ mice, as previously shown [3], or in DTX-treated *DTR-Trpm8*^*-/-*^ mice (Figure 4E,F). Thus when taken together, these data indicate that *Cgrpα-DTR*^*+/-*^ mice and *DTR-Trpm8*^*-/-*^ mice are less sensitive to noxious heat following CGRPα sensory neuron ablation; however, only *Cgrpα-DTR*^*+/-*^ mice (which have functional TRPM8) show enhanced sensitivity to cold stimuli post ablation. These data suggest that the enhanced cold responses following CGRPα sensory neuron ablation are TRPM8-dependent.

**Figure 5:**
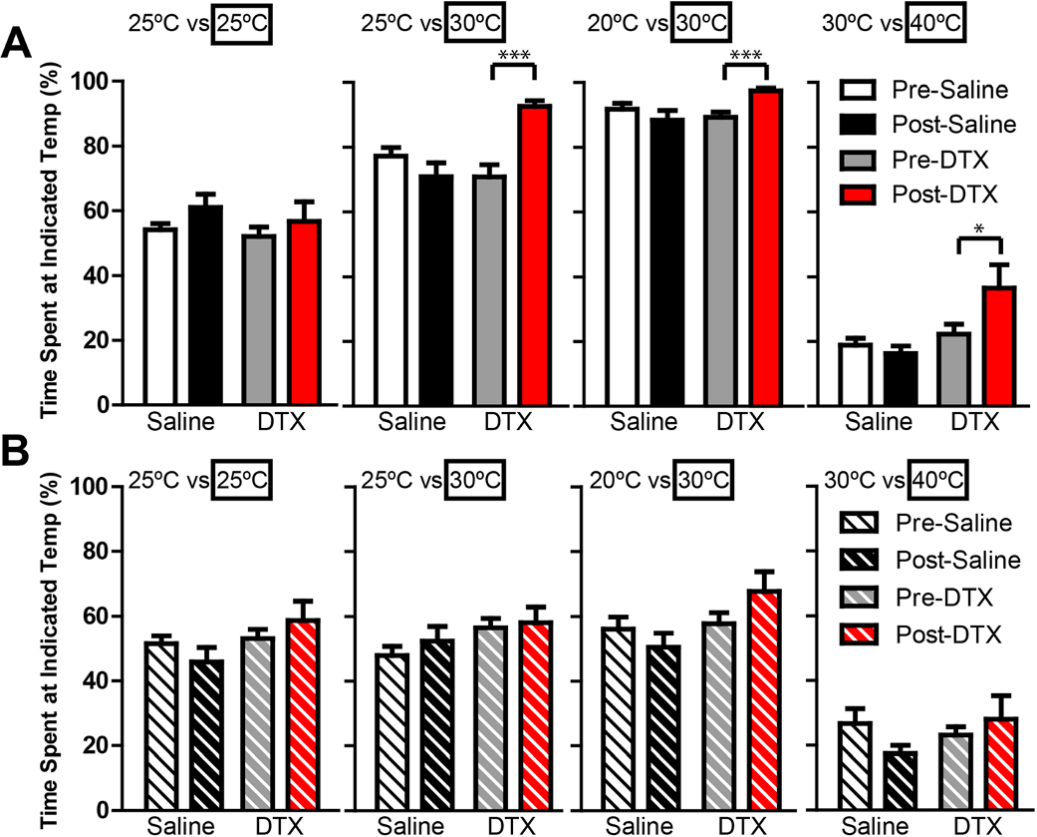
**Preference for warmer temperatures in CGRPα sensory neuron-ablated mice is dependent on TRPM8.** Temperature discrimination assay in saline- and DTX-treated **(A)** *Cgrpα-DTR* ^*+/-*^ and **(B)** *DTR-Trpm8* ^*-/-*^ mice. Floor temperature on each side was maintained at 25°C versus 25°C, 25°C versus 30°C, 20°C versus 30°C, or 30°C versus 40°C. Time spent on each side was measured over a 10 min period and expressed as a percentage relative to the boxed temperature. All values are represented as mean ± SEM. t-tests were used to compare time points between pre-saline/DTX and post-saline/DTX-treated mice. *p < 0.05, ***p < 0005.

We previously found that CGRPα sensory neuron-ablated mice showed increased preference for warm temperatures/enhanced avoidance of cooler temperatures in two-temperature discrimination assays (this assay does not distinguish whether the behavioral responses reflect increased preference or enhanced avoidance of a temperature stimulus). To determine if this altered temperature preference was TRPM8-dependent, we monitored the amount of time *Cgrpα-DTR*^+/-^ and *DTR-Trpm8*^*-/-*^ mice spent on two metal plates maintained at various temperatures (25°C versus 25°C, 25°C versus 30°C, 20°C versus 30°C, and 30°C versus 40°C; Figure 5). Neither genotype showed a preference for a side when the temperatures were the same temperature pre- or post-ablation (25°C versus 25°C; Figure 5A,B), as expected. When the plate temperatures differed, the DTX-treated *Cgrpα-DTR*^*+/-*^ mice spent more time on the warmer side relative to saline-treated controls (Figure 5A), reproducing our previous findings [3]. In contrast, the DTX-treated *DTR-Trpm8*^*-/-*^ did not differ from saline-treated controls (Figure 5B). These data suggest that the increased preference for warm temperatures/enhanced avoidance of cooler temperatures following CGRPα neuron ablation is TRPM8 dependent.

### Weight loss and thermoregulatory deficits following CGRPα sensory neuron ablation are TRPM8 independent

**Figure 6:**
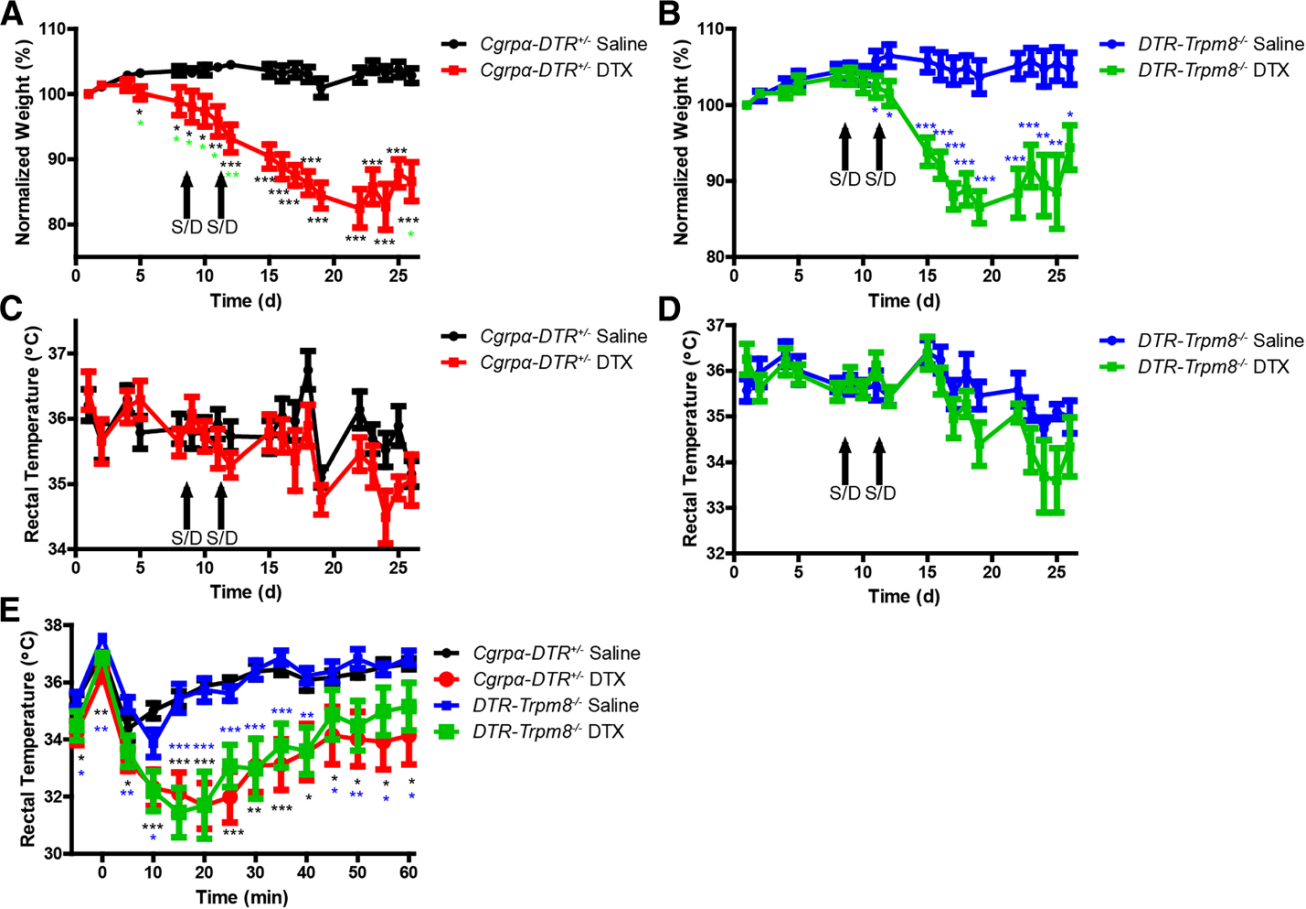
**Ablation of CGRPα-expressing DRG neurons had similar effects on body weight and thermoregulation in Cgrpα-** ***DTR*** ^***+/-***^ **and** ***DTR-Trpm8*** ^***-/-***^ **mice. (A and B)** Body weight and **(C and D)** rectal temperatures of *Cgrpα-DTR* ^*+/-*^ and *DTR-Trpm8* ^*-/-*^ mice before and after saline/DTX treatment. **(E)** The water repulsion was performed 7 days after saline/DTX injection. Mice were immersed in a 37°C water bath for 2 min. Body weight was measured every 5 min., starting before immersion and recorded every 5 min afterwards for 60 min. All values are represented as mean ± SEM. t-tests were used to compare time points between pre-saline/DTX and post-saline/DTX-treated mice. *p < 0.05, **p < 0.005, ***p < 0005. Asterisks colors for DTX-treated experimental groups are relative to saline-control groups.

We previously found that CGRPα sensory neuron-ablated mice gradually lose weight and have difficulty with thermoregulation following an evaporative cooling challenge [3]. TRPM8 contributes to body temperature regulation [13, 14], so we next sought to determine if these body weight and thermoregulatory phenotypes in CGRPα sensory neuron-ablated mice were TRPM8 dependent. To test this possibility, we monitored body weight and core body temperature of *Cgrpα-DTR*^*+/-*^ and *DTR-Trpm8*^*-/-*^ mice before and after treatment with saline or DTX. Mice from both genotypes showed significant weight loss after DTX treatment relative to saline-treated mice (Figure 6A,B). However, there were no significant differences between *Cgrpα-DTR*^*+/-*^ and *DTR-Trpm8*^*-/-*^ mice, suggesting the weight loss phenotype was independent of TRPM8 activity. Additionally, we observed no change in core body temperature in either genotype following DTX treatment (Figure 6C,D).

Next, we placed mice from each genotype in a 37°C water bath for 2 min and measured rectal body temperature at 5 min increments for 60 min (Figure 6E). Immersing the mouse in the water bath increased core body temperature, which is followed by a decrease in body temperature because of evaporative cooling. Both the DTX-treated *Cgrpα-DTR*^*+/-*^ mice and DTX-treated *DTR-Trpm8*^*-/-*^ mice had difficulty with thermoregulation, as evidenced by a greater drop in core body temperature following evaporative cooling and a slower return to baseline relative to saline-injected mice (Figure 6E). Since there were no significant differences in rectal temperature between *Cgrpα-DTR*^*+/-*^ mice and *DTR-Trpm8*^*-/-*^ mice post DTX-treatment, ablation of CGRPα neurons appears to impair thermoregulation independent of TRPM8. Note there was no significant difference between saline-treated genotypes (effectively wild-type versus *Trpm8*^*-/-*^), indicating that loss of TRPM8 does not impair thermoregulation in this evaporative cooling assay.

**Figure 7:**
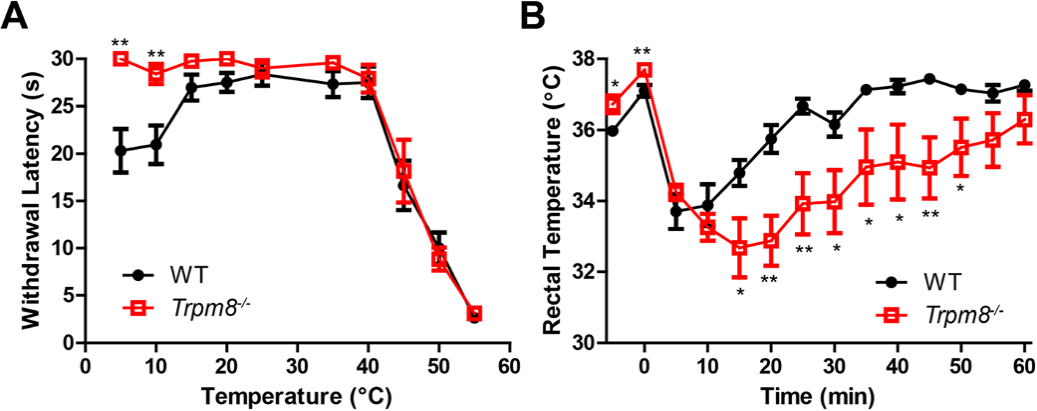
**Behavioral responses in** ***Trpm8*** ^***-/-***^ **mice lacking additional genetic modifications.** (A) WT and *Trpm8* ^*-/-*^ mice were tested in the **(A)** cold plantar and **(B)** evaporative cooling assay. All values are represented as mean ± SEM. t-tests were used to compare between genotypes at each time point. *p < 0.05, **p < 0.005.

Lastly, to determine if the sensory responses we observed in mice with genetic alterations at three loci (*Calca*, *Advillin*, and *Trpm8*) were similar to *Trpm8*^*-/-*^ mice alone, we performed many of the same behavioral experiments, except comparing WT mice to *Trpm8*^*-/-*^ mice (Figure 7; Additional file 1). These data demonstrate that *DTR-Trpm8*^*-/-*^ and *Trpm8*^*-/-*^ mice respond similarly in a variety of behavioral assays, and that genetic manipulation of *Calca* and *Advillin* did not alter TRPM8 function.

## Conclusion

We previously found that ablation of CGRPα-containing DRG neurons in mice resulted in decreased sensitivity to noxious heat [3], presumably because TRPV1 is expressed in ~50% of CGRP^+^ DRG neurons [3] and at least half of all TRPV1 neurons were ablated following DTX injection. This decreased sensitivity to noxious heat was coupled with enhanced sensitivity to noxious and innocuous cold. Here, we found that this enhanced sensitivity to cold stimuli was dependent on TRPM8, as enhanced cold sensitivity was lost when CGRPα sensory neurons were ablated in a *Trpm8*^*-/-*^ background. In contrast, both *Cgrpα-DTR*^*+/-*^ and *DTR-Trpm8*^*-/-*^ mice showed reduced responses to noxious and innocuous heat stimuli and equivalent responses following an evaporative cold challenge, showing that the heat phenotype and thermoregulatory phenotype following CGRPα sensory neuron ablation is independent of TRPM8. Our data thus strongly support the importance of TRPM8 in mediating enhanced cold sensory responses when myelinated and unmyelinated CGRPα sensory neurons are ablated.

Intriguingly, when humans underwent nerve compression to inhibit nerve impulse conduction in myelinated fibers, stimuli that were perceived as cool or cold prior to nerve compression felt burning hot and painful [15–17]. These findings suggested that loss of myelinated fiber inputs can centrally enhance cold sensation in humans, transforming cold into a stimulus that is perceived as painful. Our findings in mice may thus have parallels to human sensory biology.

## Methods

### Animal care and use

All vertebrate animals and procedures used in this study were approved by the Institutional Animal Care and Use Committee at The University of North Carolina at Chapel Hill. Mice were maintained on a 12 h:12 h light:dark cycle, were fed DietGel 76A (72-03-502; clearH2O.com) and water *ad libitum*, and were tested during the light phase. Mice were acclimated to the testing room, equipment and experimenter 1-3 days prior to testing. *Cgrpα-DTR*^*+/-*^ mice were generated by crossing *Cgrpα-GFP*^*-/-*^ female mice [7] with *Advillin-Cre*^*-/-*^ male mice [4]; and hence are heterozygous for the *Advillin-Cre* and *Cgrpα-DTR* alleles. *DTR-TRPM8*^*-/-*^ mice were generated as follows: *Trpm8*^*-/-*^ mice (The Jackson Laboratory, B6.129P2-*Trpm8*^*tm1Jul*^/J; stock #008198) were crossed with *Cgrpα-GFP*^*-/-*^ mice [7] and with *Advillin-Cre*^*-/-*^ mice [4] to generate heterozygous mice. The heterozygous offspring were crossed to generate *Cgrpα-GFP*^*-/-*^*:Trpm8*^*-/-*^ or *Advillin-Cre*^*-/-*^*:Trpm8*^*-/-*^ mice. *Cgrpα-GFP*^*-/-*^*:Trpm8*^*-/-*^ female mice were bred with *Advillin-Cre*^*-/-*^*:Trpm8*^*-/-*^ male mice, to generate mice with the following alleles *Cgrpα-GFP*^*+/-*^*:Advillin-Cre*^*+/-*^*:Trpm8*^*-/-*^*.* Diphtheria toxin (DTX, List Biologicals) was injected as described previously [3], and does not affect sensory responses or body temperature when administered to wild-type mice, as previously described [3].

### Histology

Male mice (10 weeks old) were perfused with cold 4% paraformaldehyde in 0.1 M phosphate buffer, pH 7.4. L4 dorsal root ganglia were removed from each animal and post-fixed in the same fixative for 3.5 h. One DRG from each pair was sectioned at 20 μm and collected onto Superfrost Plus slides. Every fourth section from each DRG was immunostained for NeuN (mouse IgG_1_; Millipore, MAB377, 1:250), CGRP (sheep IgG; Enzo Life Sciences, BML-CA1137, 1:300), and NF200 (rabbit IgG; Sigma, N4142, 1:800). Donkey anti-sheep IgG – Alexa-568 and a donkey anti-rabbit IgG – Alexa-647 (Invitrogen) were used at 1:200; a rat anti-mouse IgG_1_ – FITC (Invitrogen) was used at 1:10. A high-salt (2.7% NaCl) 0.05 M Tris-buffer containing 0.3% Triton X-100, pH 7.6 was used for all steps, except for final rinses in phosphate buffered saline before adding coverslips. All DRG sections were imaged on a Zeiss LSM 510 confocal microscope.

### Behavior

For all behavior experiments, 8-12 week old male mice were used. Heat sensitivity was measured by heating each hindpaw once per day using the Plantar Test apparatus (IITC) with a cut-off time of 20 s. For the tail immersion assay, each mouse was gently restrained in a towel, and the distal one-third of the tail was immersed into a water bath heated to 46.5°C or 49°C or into 75% ethanol cooled to -10°C [18]. The latency to flick or withdraw the tail was measured once per mouse. The cut-off was set at 40 s, 30 s, and 60 s, respectively. For the hot plate test, the latency to jump, shake, or lick a hindpaw was measured within a 30 s cut-off. To measure mechanical sensitivity, we used an electronic von Frey apparatus (IITC) with semi-flexible tips. Two measurements for each hindpaw were taken and averaged to determine the paw withdrawal threshold in grams. The cotton swab assay (innocuous mechanical) was performed as described [19]. For the acetone test [10], each mouse was placed into a Plexiglas chamber with a wire mesh floor, 50 μL of acetone was placed onto the glabrous surface of the left hindpaw, and the time spent licking was measured for 1 min. Icilin (60 μg in 25 μL injection volume) was injected into one hindpaw, and the number of flinches in 10 min was counted. The cold plantar assay was performed with mice resting on the glass surface of the Plantar Test apparatus (IITC) [20]. For the two-temperature discrimination assay, each mouse was placed into a Plexiglas chamber covering two metal surfaces that were set at the same or different temperatures [10, 11]. The amount of time mice spent on each side over a 10 min period was recorded. Hot and cold sensitivities were assessed on a metal plate heated/cooled to a range of temperatures (5-55°C), with a cut-off time of 30 s, as described [21]. For the water repulsion assay [22], the mouse was immersed in a 37°C water bath for 2 min. The mouse was removed from the water and placed onto a paper towel for 5 s, then its rectal temperature (deep body temperature, Tb, measured using a digital thermometer, Acorn Temp TC Thermocouple) was measured every 5 min for 60 min. at approximately the same time each day.

## Electronic supplementary material

Additional file 1: **Quantification of noxious heat-related and cold behavior assays in WT and** ***Trpm8*** ^***-/-***^ **mice.** These data are presented in a table to compare WT and *Trpm8* ^*-/-*^ mouse behavior in a variety of assays. (DOCX 16 KB)
