# Supplementary material for: Enhanced behavioral responses to cold stimuli following CGRPα sensory neuron ablation are dependent on TRPM8
Source: Mol Pain. 2014 Nov 19;10:69. doi: 10.1186/1744-8069-10-69 (PMC4247560; doi:10.1186/1744-8069-10-69)
Supplement: Supplementary file 1 — Additional file 1: Quantification of noxious heat-related and cold behavior assays in WT and Trpm8 -/- mice. These data are presented in a table to compare WT and Trpm8 -/- mouse behavior in a variety of assays. (DOCX 16 KB) [file 12990_2014_674_MOESM1_ESM.docx]

**Additional File 1. Quantification of noxious heat-related and cold behavior assays in WT and *Trpm8^-/-^* mice.**

| **Behavior** | **Response** |
| --- | --- |
| ***HEAT*** |  |
| **Tail Immersion (46.5°C)** | Latency to Flick (s) |
| WT | 31.5 ± 2.4 |
| *Trpm8^-/-^* | 29.8 ± 2.6 |
| **Tail Immersion (49°C)** | Latency to Flick (s) |
| WT | 7.0 ± 0.7 |
| *Trpm8^-/-^* | 8.2 ± 1.0 |
| **Hot Plate (52°C)** | Withdrawal Latency (s) |
| WT | 24.0 ± 2.0 |
| *Trpm8^-/-^* | 24.6 ± 0.4 |
| **Hargreaves** | Withdrawal latency (s) |
| WT | 8.6 ± 0.5 |
| *Trpm8^-/-^* | 8.4 ± 0.6 |
| ***COLD*** |  |
| **Acetone** | Time Spent Licking (s) |
| WT | 3.9 ± 0.5 |
| *Trpm8^-/-^* | 1.3 ± 0.3** |
| **Tail Immersion (-10°C)** | Latency to Flick (s) |
| WT | 44.3 ± 4.2 |
| *Trpm8^-/-^* | 59.0 ± 1.0** |
| **Cold Plantar** | Withdrawal Latency (s) |
| WT | 10.5 ± 0.6 |
| *Trpm8^-/-^* | 20.0 ± 0.0*** |
| ***MECHANICAL*** |  |
| **Cotton Swab** | Withdrawal Frequency (%) |
| WT | 56.0 ± 5.5 |
| *Trpm8^-/-^* | 51.1 ± 6.3 |
| **Von Frey** | Withdrawal Threshold (g) |
| WT | 7.0 ± 0.3 |
| *Trpm8^-/-^* | 7.7 ± 0.4 |

n = 10 mice/group, **p < 0.005, ***p < 0.0005.
